# Decision-making on intra-household allocation of bed nets in Uganda: do households prioritize the most vulnerable members?

**DOI:** 10.1186/1475-2875-13-183

**Published:** 2014-05-17

**Authors:** Yukyan Lam, Steven A Harvey, April Monroe, Denis Muhangi, Dana Loll, Asaph Turinde Kabali, Rachel Weber

**Affiliations:** 1 Johns Hopkins Bloomberg School of Public Health, 615 N. Wolfe Street, Baltimore, MD 21205, USA; 2 Johns Hopkins Bloomberg School of Public Health Center for Communication Programs, 111 Market Place, Suite 310, Baltimore, MD 21202, USA; 3 Makerere University, School of Social Sciences, P.O. Box 7062, Kampala, Uganda; 4 Team Initiatives Ltd, P.O. Box 3963, Teachers House, Plot 28/30 Bombo Road, Kampala, Uganda

**Keywords:** Malaria, Insecticide-treated bed nets (ITNs), Bed net allocation, Bed net access, Mosquito nets, Uganda, Qualitative research, Net use patterns

## Abstract

**Background:**

Access to insecticide-treated bed nets has increased substantially in recent years, but ownership and use remain well below 100% in many malaria endemic areas. Understanding decision-making around net allocation in households with too few nets is essential to ensuring protection of the most vulnerable. This study explores household net allocation preferences and practices across four districts in Uganda.

**Methods:**

Data collection consisted of eight focus group discussions, twelve in-depth interviews, and a structured questionnaire to inventory 107 sleeping spaces in 28 households.

**Results:**

In focus group discussions and in-depth interviews, participants almost unanimously stated that pregnant women, infants, and young children should be prioritized when allocating nets. However, sleeping space surveys reveal that heads of household sometimes receive priority over children less than five years of age when households have too few nets to cover all members.

**Conclusions:**

When asked directly, most net owners highlight the importance of allocating nets to the most biologically vulnerable household members. This is consistent with malaria behaviour change and health education messages. In actual allocation, however, factors other than biological vulnerability may influence who does and does not receive a net.

## Background

Ownership of insecticide-treated bed nets (ITNs) has increased greatly in recent years. In Uganda, the percentage of households owning at least one ITN grew from 16% to 60% between 2006 and 2011. Despite these gains, only 45% of the country’s population had access to an ITN in 2011 [1]. As defined by the Demographic and Health Survey (DHS) and as used in this paper, ‘access’ refers to the percentage of the population living in households with one ITN for every two people. Thus, the gap derives both from households with no ITNs and from those with too few ITNs to cover all household members. In fact, only 28% of households had at least one ITN for every two people. Both peri-urban and rural households owned an average of 1.3 ITNs, while their average size was 3.8 and 5.1 persons, respectively [1].

Moreover, recent research by Killian *et al.* suggests that current methodology significantly overestimates bed net access [2]. The access problem is compounded by the fact that ITNs – including long-lasting insecticidal nets (LLINs) – wear out at varying rates that depend on household environment, as well ask net use, care, and repair [3-7]. At the same time, household poverty and dependence on global donor funds affect ability to replace nets readily [8,9].

Pregnant women, infants, and young children are particularly vulnerable to malaria [10,11]. Studies in sub-Saharan Africa have found that ITN use contributes to significant reductions in placental malaria, low birth weight, and still-births, as well as reduced morbidity and mortality among children under five [12,13]. Continuing net shortages make it critical to understand how households allocate nets among their members and whether the most biologically vulnerable groups are protected.

There have been few studies on intra-household net use patterns. Among the more recent research, two population-based surveys concluded that households with insufficient nets, or insufficient ITNs, to cover all members give priority to pregnant women and children under five [14-18]. The first, a multi-country study in Ethiopia, Ghana, Mali, Nigeria, Senegal, and Zambia, used survey data from 2000 to 2004 about households, their members, and the occupants of each bed net owned [14]. In each country, data showed that children under five and women of reproductive age, especially pregnant women, were most likely to sleep under a net. Children between five and 14 years of age and adult males were least likely. However, sub-analysis by study site showed that some areas had different net use patterns.

The second study, conducted in 2006, analysed data from the Tanzania National Voucher Scheme, which offered pregnant women subsidies to purchase ITNs [16]. Surveys enumerated which household members slept under each net the previous night and which slept without a net [16,19]. Researchers studied intra-household “net equity” by determining who slept under treated versus untreated nets and who slept under nets with fewer holes [16]. Results showed that infants were the group most likely to sleep under an intact ITN. In households with at least one untreated net, one ITN, and one infant or young child, probability of ITN use decreased by age: infants had the highest probability, followed by young children and women of reproductive age, adult males and older children, and finally older women.

A Ugandan study using 2000–2001 DHS data found that children were much more likely to sleep under a bed net if their mothers also used one [17]. Although the DHS did not ask about the number of nets in the household or whether children shared a sleeping space with their mother, stratified statistical analyses strongly suggested that children who slept under a bed net did so because they were sleeping in the same space as their mother [17]. Researchers thus concluded that primary protection was not aimed specifically at children themselves [17].

The present study used qualitative methods to examine intra-household net allocation in four Ugandan districts. It explored hypothetical versus actual allocation and compared both to international guidelines that prioritize pregnant women and children under five [20]. The generic terms, “net” and “bed net,” are used rather than “ITN” or “LLIN,” due to difficulty in conclusively determining net type during household visits. Use of the terms, “ITN” and “LLIN,” imply explicit information on the type of net being referenced.

## Methods

This article presents data from the first two phases of a three-phase study on the culture of ITN use in Uganda. Phase 1, conducted early in the rainy season in March 2012, began with Nebbi and Luwero Districts in Uganda’s Northern and Central Regions, respectively. In each district the study team recruited six households: three rural and three peri-urban. Study team members completed sleeping space questionnaires (SSQs) for every sleeping space in each household and conducted an in-depth interview (IDI) with one adult household member. The team also organized one rural and one peri-urban focus group discussion (FGD) per district, with participants drawn from both participating and non-participating households.

**Figure 1:**
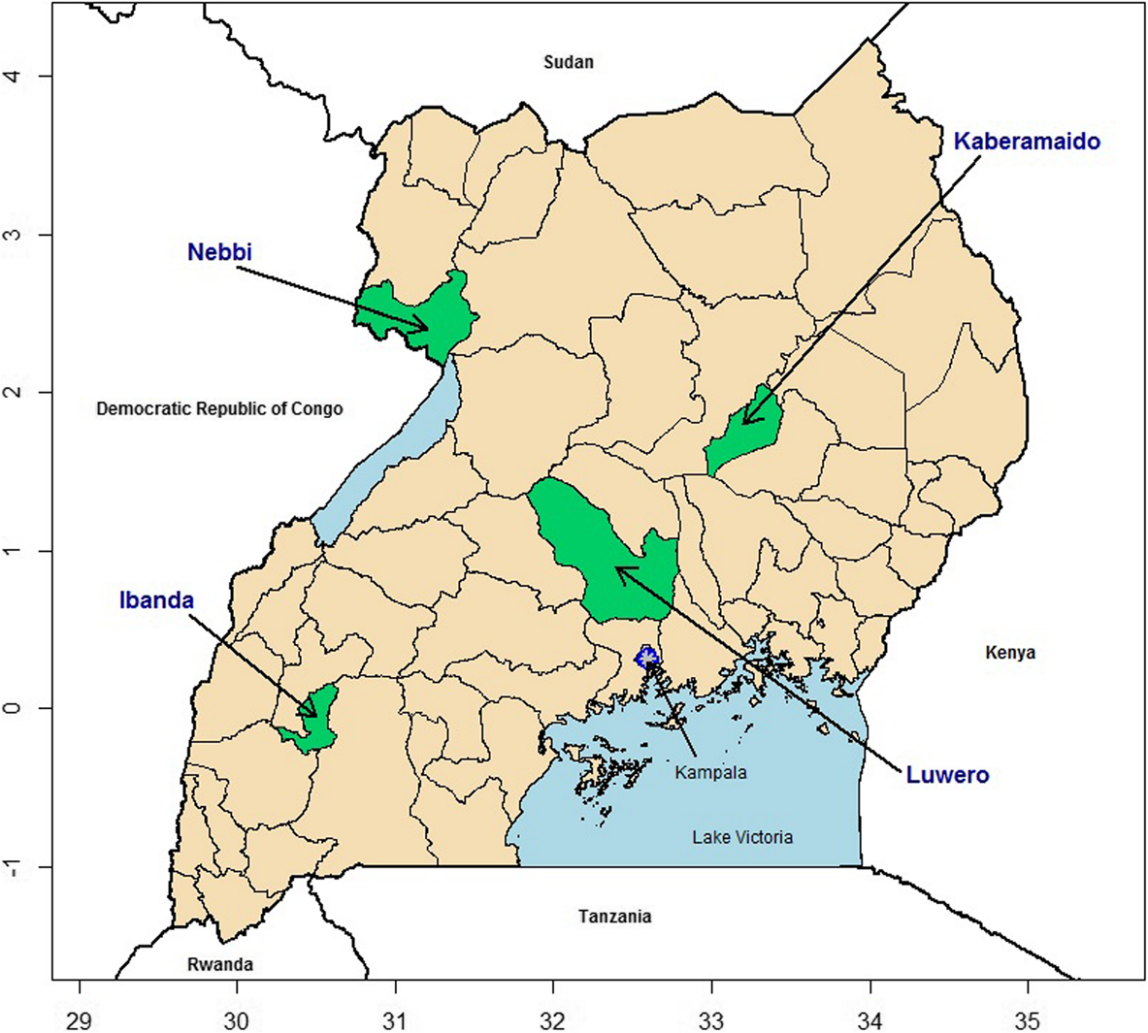
Map of Uganda with study areas highlighted in green.

In phase 2, conducted during the dry season in January 2013, the team revisited the 12 households from Nebbi and Luwero and updated sleeping space information by administering new SSQs. They also enrolled two new households in each district and completed SSQs and IDIs for them. In addition, Ibanda and Kaberamaido Districts were added in the Western and Eastern Regions, respectively, with six households recruited from each district. IDIs were also conducted with these households. However, as IDIs in phase 2 did not address net allocation, their findings are not presented here. Two FGDs were conducted in each new district with participants belonging to already recruited households or other households from the same sub-counties (Figure 1).

### Sampling

The four districts were purposively chosen to represent Uganda’s four regions and to include districts where nets had been distributed in the preceding three years. Within each district, the district chairman or district health officer helped select two communities, one from a peri-urban and another from a rural sub-county. In each community, village leaders assisted with purposively selecting households for SSQs and IDIs to ensure geographic representation and ownership of at least one bed net.

Within households, any adult member was eligible to complete the SSQ. Most often, the participants sleeping in a given sleeping space assisted with the SSQ for that space. IDIs were conducted with the head of household or his or her spouse when possible. An adult household member not participating in the IDI was eligible to partake in a FGD. Additional FGD participants were recruited by convenience sampling from households owning at least one bed net in the same village. Participants, generally the head of household or spouse, were selected based on gender such that one all-male and one all-female FGD, each consisting of seven to ten participants, was conducted per district. All IDIs and FDGs were audio recorded.

### Interview and focus group themes

**Figure 2:**
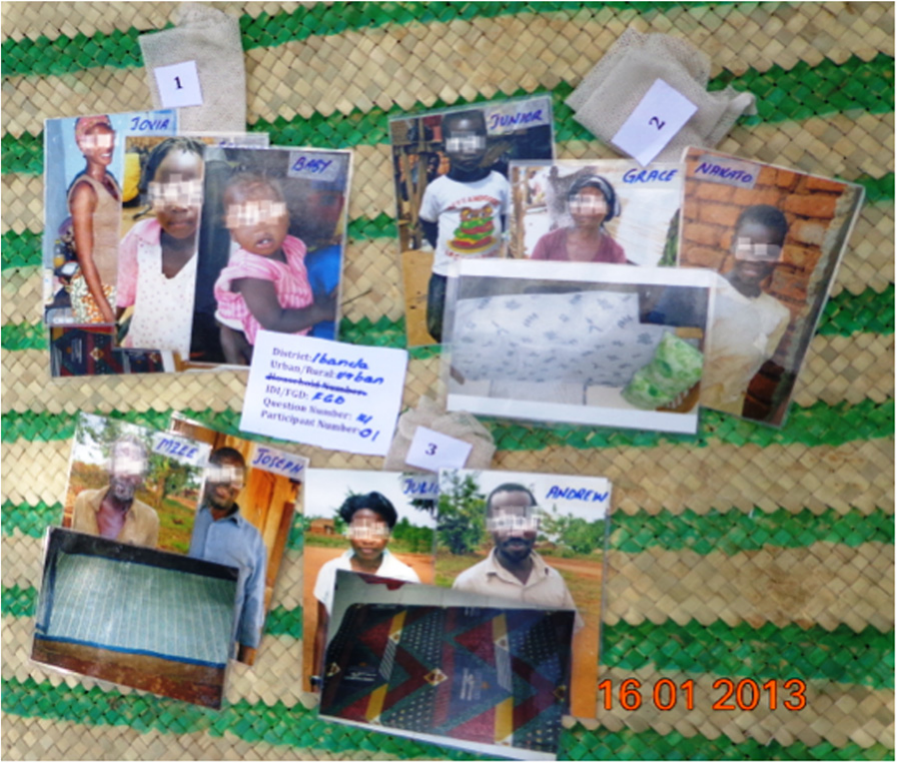
Sleeping space arrangement recorded from Ibanda peri-urban focus group.

All FGDs and the phase 1 IDIs included the topics of malaria prevention, use and care of bed nets, and household sleeping space allocation. They also included a participatory exercise designed to elicit factors that influence intra-household allocation of sleeping spaces and bed nets. In this exercise, moderators provided participants with photographs of ten different individuals, separate photographs of four sleeping spaces, and three swatches of bed net fabric. The subjects in the photographs included men, women, boys, and girls ranging from infant to elderly. Two of the pictured sleeping spaces consisted of beds with spring mattresses and linens, another was a foam rubber mattress placed on the floor, and the last was a thin straw mat on the floor. One of the bed net swatches represented a new net with no holes, another represented a slightly used and discoloured net with a few small holes, and the third a very worn and soiled net with several large holes and tears (Figure 2). Materials used for the participatory exercise (photographs and swatches) were the same between phases 1 and 2.

During the allocation exercise, the IDI or FGD participants were asked to assign the individuals to sleeping spaces and bed nets. Since there were only three nets, one of the participant’s tasks was to decide which sleeping space would remain without a net. The moderator then asked the participant to explain the factors influencing his or her assignments. In the focus group setting, after one volunteer presented his or her arrangement, the moderator invited discussion from other group members. The moderator then invited two additional participants to complete the exercise and suggest alternative arrangements. Each configuration was photographed.

### Sleeping space questionnaires

The study team completed SSQs for each sleeping space in the selected households. A study team member asked who had slept in each space the previous night, as well as each occupant’s age, sex, marital and pregnancy status, education level and relationship to the household head. The questionnaire also elicited characteristics of any net associated with the sleeping space, including how long it had been used, how it was cared for, and whether it had been repaired. Some of the questions, such as whether the net appeared dirty, were verified by observation. However, most of the care and repair questions, such as how often the net was washed, were not.

### Analysis

IDIs and FGDs were transcribed, translated into English, and coded for key themes using ATLAS.ti [21]. A codebook developed by two members of the research team included codes relevant to the sleeping space exercise and to sleeping space and net allocation more broadly. Stata® version 12 was used to generate descriptive statistics from the sleeping space questionnaires [22]. Also using SSQ data, sleeping space maps were manually constructed to analyse sleeping arrangements at the household level.

### Ethical review

Ethical approval for the research was secured from the Johns Hopkins University Bloomberg School of Public Health Institutional Review Board in Baltimore, Maryland, and from the Joint Clinical Research Center and the Uganda National Council for Science and Technology Institutional Review Boards in Uganda.

## Results

In phases 1 and 2, a total of eight focus groups were conducted, one male and one female in each of the four districts. Each group included seven to ten participants. Phase 1 also incorporated 12 IDIs, six in Luwero and six in Nebbi (Table 1). As noted earlier, phase 2 IDIs did not address net allocation so their results are not reported here. The sleeping space allocation exercise with photographs was carried out during FGDs in both phases and during IDIs in phase 1. SSQs were administered for a total of 107 sleeping spaces in 28 households: six each in Ibanda and Kaberamaido; eight each in Luwero and Nebbi (Table 2). In each district, half of the households were located in peri-urban areas, the other half in rural.

**Table 1:** In-depth interviews and focus group discussions by district

**District**	**In-depth interviews**			**Focus groups (participants)**		
	**Male**	**Female**	**Total**	**Male**	**Female**	**Total**
Ibanda	NA	NA	NA	1 (10)	1 (10)	2 (20)
Kaberamaido	NA	NA	NA	1 (10)	1 (10)	2 (20)
Luwero	2	4	6	1 (9)	1 (7)	2 (16)
Nebbi	2	4	6	1 (8)	1 (10)	2 (18)
**Total**	**4**	**6**	**12**	**4 (37)**	**4 (37)**	**8 (74)**

**Table 2:** Sample of households and sleeping spaces, by district

**District**	**Households surveyed**	**Average no. of members per household**	**No. of sleeping spaces (% of total)**	**Average no. of sleeping spaces per household**	**Average no. of occupants sleeping together in a single space the prior night**
Ibanda	6	7.0	28 (26.2%)	4.7	1.6
Luwero	8	5.5	30 (28.0%)	3.8	1.7
Kaberamaido	6	6.3	24 (22.4%)	4.0	1.6
Nebbi	8	6.4	25 (23.4%)	3.1	2.4
**Total**	**28**	**6.3**	**107**	**3.8**	**1.8**

All 28 households owned and used at least one net (Table 3). Eleven (39%) had nets associated with every sleeping space, while 17 (61%) had one or more sleeping spaces with no net. Hereafter this group is referred to as the ‘17-household subsample with insufficient nets.’

**Table 3:** Shortage of bed nets within households, by district

**District**	**Total no. of sleeping spaces**	**Sleeping spaces without bed nets**	**Percentage of sleeping spaces without bed nets**
Ibanda	28	16	57.1%
Luwero	30	9	30.0%
Kaberamaido	24	5	20.8%
Nebbi	25	9	36.0%
**Total**	**107**	**39**	**36.5%**

Of the 107 total sleeping spaces, 68 (64%) had nets while 39 (36%) did not. Among the 68 with nets, 58 (85%) were protected by a single net while nine had an extra, secondary net. Information about one space was ambiguous. Two of the spaces with extras nets were located in households where some *other* sleeping space lacked a net. As reported by respondents, the nets ranged in age from one month to five years, with an average of 26 months. Age information was missing for four nets.

Across the 28 households, there were 175 individuals who slept in the household the previous night, yielding an average of 6.25 members per household. Table 4 shows ages for all but four of those 175 individuals. The 107 sleeping spaces yielded an average of 3.82 sleeping spaces per household. Ten of these 107 spaces were unoccupied the night prior to the survey. Excluding these ten, each sleeping space averaged 1.8 occupants.

**Table 4:** Household members sleeping in house on prior night, by age group

**Age group**	**Number of members**	**Percentage of total**
Under 5 years old	39	22.3%
5-14 year olds	58	33.1%
15-49 years old	64	36.6%
Over 49 years old	10	5.7%
Unknown	4	2.3%
**Total**	**175**	**100%**

### Hypothetical allocation: the sleeping space and net allocation exercise

During in-depth interviews and focus group discussions, participants generally agreed that pregnant women and very young children should receive priority when allocating sleeping spaces and nets. In the hypothetical net allocation activity that used photographs, the pregnant woman and the children meant to appear under five years old were most often assigned a bed with a mattress and the new net with no holes. Across all districts and among participants of both genders, the baby was most often assigned the best net, followed by the pregnant woman (see, for example, the arrangement portrayed in Figure 2). The old man was least likely to be assigned the best net and often was assigned no net at all. On only a few occasions did participants assign him the best net and sleeping space. Opinions differed regarding the age at which older adults might need additional protection. There was also considerable variation in placement of older children, whose assignments ranged from the best net and sleeping space to the worst.

Participants gave multiple reasons for their allocation decisions. However, the justification given almost universally for prioritization was biological vulnerability. For example, after assigning the pregnant woman, baby, and youngest boy and girl to the best bed and newest net, one participant explained her arrangement as follows:

“This woman is pregnant and these children are still young, so they need proper care so that they are safe from malaria. That’s why they have to use a treated mosquito net, and again they have weaker blood which makes it easier for them to get malaria.”

[Peri-urban FGD, Kaberamaido]

Many participants gave similar reasons – including risk of losing the pregnancy – for prioritizing the same individuals: *“She is pregnant and if she is bitten by a mosquito, malaria will also spread to the unborn baby, and this may lead her to have a miscarriage.”* [Rural FGD, Ibanda] Others stated that pregnant women and very young children would be unable to defend themselves by chasing away or killing mosquitoes. Children over age five were sometimes perceived as less vulnerable, but still at risk: *“They are also young but can resist malaria, unlike the young one who is very vulnerable to malaria.”* [Rural FGD, Kaberamaido]

While participant opinions differed about net allocation to older children and adults, those perceived as having “good health” or “very strong immune system[s]” were not assigned the best net. [Rural and peri-urban FGDs, Ibanda] In explaining why the two oldest men in the photographs were often left without a net, many participants voiced the sentiment that *“old people… cannot be disturbed by malaria like other people. Even if they are bitten by mosquitoes and they fall sick, they do not get disturbed like how these other people suffer.”* [Peri-urban FGD, Ibanda] A female participant from Luwero echoed this logic:

“I would not give first priority to the youth because they are strong and their bodies can resist malaria… I would only consider those that are more vulnerable and leave those that are strong enough to fight.”

[Rural IDI, Luwero]

Beyond physical vulnerability, participants sometimes based their decisions on personal characteristics they attributed to the pictured individuals. For example, some participants denied the old man a net because they perceived him as someone who did not care about his well-being, because he was old and no longer “useful,” because he was more able to endure malaria, or because he was seen as a drunkard and thus unable to feel the mosquitoes. As one male participant explained:

“If the old man were a responsible person who cared about taking care of himself, I would have given him a good net. But from his appearance you can just see that he is someone who drinks alcohol, an indication that he does not want to take care of himself.”

[Rural FGD, Nebbi]

Alternatively, most members of another male focus group in Ibanda agreed when one participant suggested that the two oldest men pictured could do without a net because *“they can spend the whole night chatting, and when they get disturbed they can decide to smoke and the smoke can chase away the mosquitoes.”* [Rural FGD, Ibanda]

Some participants noted that several of the individuals in the photographs appeared nicely dressed or clean and used this reason to assign them better sleeping spaces and nets. Assuming the character of a well-dressed young man in one photograph, a participant stated: *“I have just separated from my home. That is why my things are still new. And again—me, I am like a youth, I should not have dirt. I need to be clean.”* [Rural FGD, Kaberamaido]

Participants also invoked the assumed economic or physical capacity of the pictured individuals. Adolescents, young adults, and middle-aged adults were described as able to care for nets, repair damaged nets, and as having the energy to work and earn money for a new net. As one male participant stated in assigning the net with many holes to three such individuals, *“if they are grown-up girls, they should be able to see that the net is old, and they can also buy a new net because they also know how to look for money.”* [Rural FGD, Kaberamaido]

Both responsibility and the burden associated with caring for people of varying ages informed allocation decisions in the hypothetical exercise, but participant interpretation of these issues varied. As one female participant explained:

“For me, if I had an old man in my family and young people, I would give a mosquito net first to the old man because if he got sick, I would still be the one to suffer with the medical bills. Treatment for old people is normally more expensive than that of young ones and, because of that, I would give the old man first priority.”

[Rural FGD, Luwero]

Another participant from the same FGD disagreed:

“I would mostly consider children below seven years to sleep under the net because when children fall sick, it is us parents who suffer treating them, and the degree of severity of malaria among children is higher than that of adults.”

A third cited the responsibility of caring for young children and other household members:

“For me I would give myself the last opportunity to get a net because being the owner of the household, I am the one who is supposed to provide my dependents with mosquito nets. If I gave myself the first priority, I would appear as if I had forgotten the others and left them to continue suffering [from mosquito bites]. It would even look bad for me to get a net when the other dependents have nothing.”

### Observed allocation: sleeping space questionnaires from the 17-household subsample with insufficient nets

**Figure 3:**
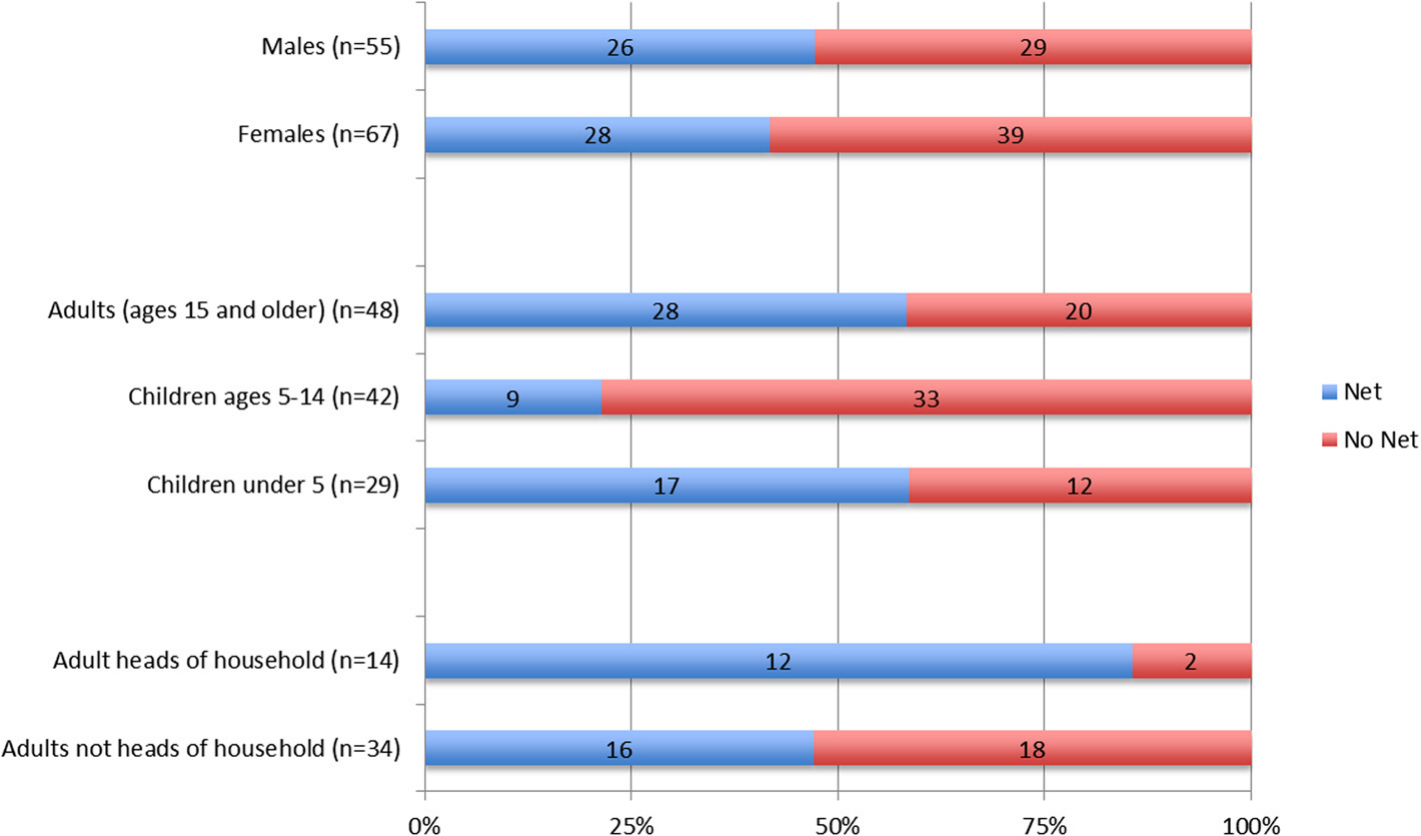
Numbers and percentages of individuals whose sleeping space had a net or no net among the 17 households with insufficient nets.

The net allocation observed in participating households differed somewhat from that suggested by participants in the hypothetical net allocation exercise. To explore intra-household prioritization of nets, data are presented from the subsample of 17 households with insufficient nets to cover all sleeping spaces. A total of 122 individuals slept in these households on the night prior to their participation in the study. Of these, 68 (56%) slept in a space with no net. When these inhabitants are stratified by age (Figure 3), 12 of 29 children under five (41%) slept with no net, as did 33 of 42 children ages 5 to 14 (79%) and 20 of 48 adults ages 15 and older (42%). There was only one pregnant woman in the subsample; she was reported to sleep with a net. Two other pregnant women in the broader sample lived in households with sufficient nets to cover all sleeping spaces.

**Figure 4:**
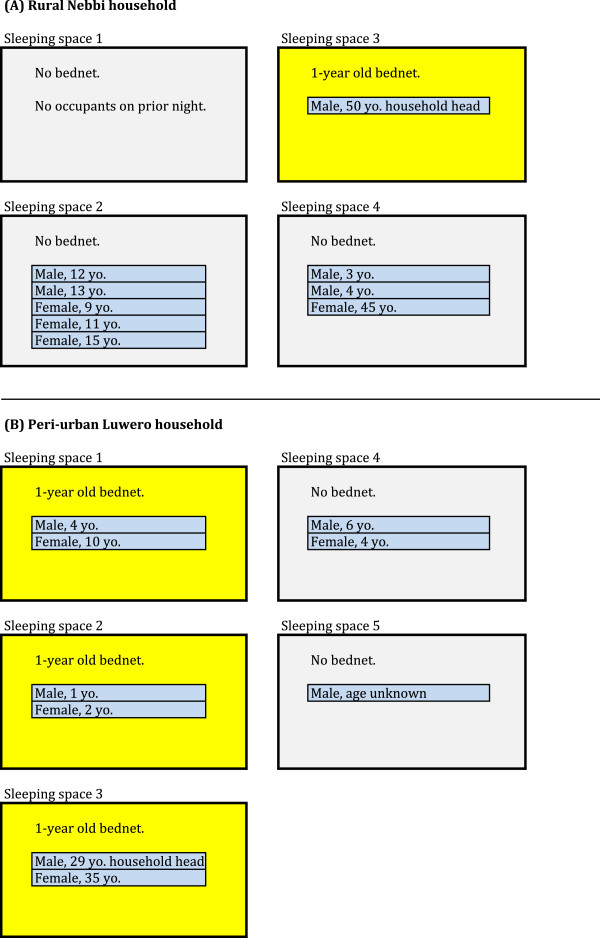
Schematic diagram of sleeping arrangements observed in two households.

In the 17-household subsample, 14 heads of household slept at home on the night prior to the SSQ. The identity and whereabouts of three heads of household were unknown. Only two out of the 14 identified heads of household (14%) slept in a space with no net, compared to 18 of 34 adults (53%) who were not heads of household (Figure 3). Of the 12 heads of household in the subsample who slept with a net, half did so while at least one child under five in their household slept without one. Figure 4A illustrates one such example. In most of these cases, the household head either slept alone or with older children or adults. In only one case did the household head share his sleeping space with a child under five. This does not necessarily imply that *all* children under five in those households slept unprotected. For instance, in one peri-urban Luwero family, the head of household and his wife slept together under one net, a 1 year-old boy and 2 year old girl shared another, a 4 year old boy and 10 year-old girl shared a third, but a 4 year-old girl and 6 year-old boy slept without a net (Figure 4B). However, there was no household in which all children under five slept with a net while the head of household was left without.

After describing how she and her infant, 4- and 6-year old children sleep together on a papyrus mat on the floor with no net while her husband sleeps on a bed with a net and a mattress, a Nebbi IDI informant offered the following explanation:

“We had in mind that the man, as the head of the family, should be the one to get it first; [he] has to sleep on the bed. If God provides more, then me and the kids shall get later.”

[Rural IDI, Nebbi]

The informant added that the single bed had room for only one person, making it impossible for her and her children to sleep on it together. On the other hand, the children were too young to sleep alone, so to be with her the only option was to sleep on the floor. As for the net, the informant reported,

“…that one is just because it is not possible to hang the mosquito net on a papyrus mat, but the net can be hung on the bed so it was appropriate for the mosquito net to be on the bed.”

Household heads were not the only ones sometimes prioritized over children under five in net allocation. In four households, older children, teenagers, or adults who were not the head of household slept with nets while at least one child under five did not. Again, this did not necessarily imply that *all* children under five slept without a net in those households.

Looking across districts, Ibanda was the district with the most households experiencing net shortage; all six households surveyed there lacked a net for at least one sleeping space. It was also the district with the most prioritization of family members other than children under five in net allocation. There were four households where children under five slept in spaces without nets while older children, adolescents, and other adults (both heads and non-heads of household) were allocated spaces with nets.

## Discussion

Malaria control programmes have emphasized the importance of targeting vulnerable groups, including pregnant women and children under five, through health communication and net distribution campaigns and other malaria prevention interventions [11,20]. The early study by Alaii *et al.* on intra-household net allocation in Kenya found that heads of household were more likely than children under five to sleep under ITNs [15]. Most subsequent studies conclude the opposite: Households usually prioritize biological vulnerability over other possible allocation criteria. For instance, after conducting IDIs with 19 adults in Zanzibar, Beer *et al.* found that young children received priority for net use when households had insufficient nets to protect all members [23]. In their survey of ITN use in over 2,400 Sri Lankan households, Fernando *et al.* found that 75% and 90% of children under five slept under a net during low and high malaria transmission season, respectively [24,25]. Baume and Marin’s five-country population-based review of net use found “women of reproductive age and children under five… were most likely to use the net; least likely were children of age five to 14 and adult males.” The authors concluded that net campaigns should focus more on year-round use than biological vulnerability [14].

Nevertheless, most studies also cite exceptions. In the Fernando study [24], fewer than 50% of pregnant women slept under a net. Baume and Marin found that 18% of Senegalese households owning a single net allocated it to someone other than a child under five or a pregnant woman. They also found that net use among children under five and pregnant women declined in Nigeria between 2000 and 2004, along with an overall decline in net use.

The results of this study are similarly equivocal, and suggest a difference between social norms and observed behaviours. Participants in the hypothetical allocation exercise made decisions mostly in line with program priorities. In assigning three nets across four sleeping spaces and ten individuals, the great majority prioritized the pregnant woman, the baby, and the children they perceived to be under five. The pregnant woman and baby almost always received the new net, while the slightly used net with a few holes tended to go to other young children. Overall, these participants seemed to understand – and at least in theory accept – the importance of protecting household members at greatest biological risk. As one female participant explained:

“When they came to distribute nets, priority was being given to pregnant women and children under five… Young children are highly vulnerable to malaria when they are bitten by mosquitoes, a case that is not the same like the old.”

[Peri-urban FGD, Ibanda]

Some participants used allocation criteria unrelated to physical vulnerability that nonetheless aligned with program priorities. For instance, while young and middle-aged adults are considered less vulnerable than children under five, IDI and FGD participants often mentioned this group’s capacity to earn money to buy nets for themselves as a rationale for assigning them lower priority. Only occasionally did participants offer a non-biological rationale contrary to program priorities, such as the FGD participant who said she would give the best net to the older man, *“because if he got sick, I would still be the one to suffer with the medical bills.”* [Rural FGD, Luwero]

The questionnaires from households with too few nets to cover all sleeping spaces reveal a more complex story. A substantial proportion—12 out of 29—of children under five in this subsample slept without a net. These 12 children were spread across nine households in the 17-household subsample. In all but one of these nine households, others were prioritized in receiving a net. In theory, these eight households could have reallocated a net from one sleeping space to another to achieve greater coverage of children under five. The lone net in the ninth household was used for a sleeping space with an infant; a four-year old slept elsewhere. Adults who received a net when children under five did not were typically heads of household sleeping alone or with an adult partner.

The fact that a household could theoretically reallocate nets to achieve greater coverage of children under five does not imply that it completely disregards young children in allocating nets. Half of the eight study households in this position were achieving partial coverage of their children under five. The data in these cases offer minimal information about the net allocation logic being used. For example, one possibility is that households allocate nets first to protect some of the young children, and then to protect the household head or others. An alternative scenario is that they first allocate nets to household heads, then see which children under five they can protect with the remaining nets. A reasonable interpretation of the data would be that children under five are not always being prioritized, that household heads and others do sometimes receive nets to the exclusion of children under five, and that in some cases this may reflect balancing protection of some of the household’s young children with protection of other household members, rather than complete disregard of all young children and malaria programme priorities.

Moreover, why some households allocate nets to less biologically vulnerable members in preference to those with greater vulnerability remains unclear. One possible explanation is economic, as some studies suggest that families allocate health resources to those *“perceived as a productive asset for the household”*[26,27]. As primary wage earners and caretakers, household heads may receive better nets and sleeping spaces. However, these studies look at health expenditures in general, not those specifically related to malaria. Further, they focus on curative rather than preventive care. One suggestion that perceived productivity may play a role in net allocation comes from a participant in the peri-urban Ibanda women’s focus group:

*“Mzee* [the old man pictured in the exercise] *and Joseph* [a man meant to appear in his late 50s] *are on the mat and with no mosquito net because they are old and no longer useful. Even if they get malaria, we won’t bother.”*

When the moderator asked if other participants agree, they responded in unison that they did. But the comment related to older men, not to young children or pregnant women.

The Nebbi participant who slept on the floor with her children while her husband slept on the bed mentioned three factors: deference to her husband’s position as household head, a bed too small for more than one person, and perceived inability to hang the net over a mat. During her interview, she mentioned that her husband had been away for some time, yet she and the children continued to sleep on the mat. The net and bed remained empty. It is not clear which of her three reasons, if any, takes precedence. Her story is consistent with Alaii *et al.*’s observation that in Kenya, *“ITNs were not readily redeployed in the face of shifting sleeping patterns”*[15]. Perhaps households see nets as belonging to a particular individual or sleeping space and thus not available for reallocation when that person is away or that bed unoccupied. To the authors’ knowledge, there is no literature on this question, and it warrants additional research. Similar factors may be at play in the two households which had an extra net for one sleeping space while another space had none.

### Limitations

Several limitations preclude definitive conclusions on the dynamics of intra-household net allocation. First, the descriptive statistics presented above cannot be generalized to the broader population since the study participants did not come from a random sample. The data indicate that the most biologically vulnerable individuals are not always prioritized, despite a relative consensus to the contrary expressed during the hypothetical exercise. However, the study was not designed to quantify the degree to which this occurs.

Second, SSQ participants were not asked explicitly about what factors influenced their actual net allocation decisions. It may be beneficial to conduct SSQs on a larger sample of households, and randomly select a proportion of these households for in-depth interviews. Even in the case of a small, non-random sample, it would be useful to explore with respondents the reasons for allocating nets as they do.

Finally, participants interpreted the hypothetical net allocation exercise in different ways. Most created stories around the people in the photographs, attributing different characteristics and familial relationships to them. This is both a strength and a weakness. On the one hand, participants drew upon these individually-crafted narratives in their allocation decisions, thus providing a wealth of information about social norms. On the other hand, the differences between the narratives limit their usefulness for drawing cross-cutting conclusions. Nonetheless, the results make clear that criteria unrelated to biological vulnerability remain important in intra-household net allocation, reflecting a need for both continued research and additional resources to improve bed net coverage within the household.

## Conclusions

This research showed that in both a hypothetical exercise and in reality, the most biologically vulnerable household members usually receive nets when there are too few nets to protect everyone. This is consistent with malaria behaviour change and health education messages. However, the observed patterns of intra-household net allocation suggest that criteria other than biological vulnerability also play an important role. More research about what specific criteria other than biological vulnerability factor into these decisions would help improve the effectiveness of messages promoting LLIN use in households with insufficient nets to cover every sleeping space. Programme managers could then design messages to accompany distribution efforts that acknowledge and address households’ concerns in this domain, rather than only stressing the vulnerability of young children and pregnant women to malaria. At the same time, the results of this study highlight the need to strengthen net distribution efforts so that households are less frequently forced to prioritize certain members over others.

## Competing interests

The authors declare that they have no competing interest.

## Authors’ contributions

YL was responsible for data analysis and interpretation as well as drafting and managing edits to the manuscript. SH participated in study design and data collection, and provided oversight and input throughout data collection and analysis. SH also made substantial contributions to drafting and revising the manuscript. AM was involved in data analysis and interpretation and made significant contributions to the manuscript. DM oversaw data collection and provided significant feedback on the manuscript. DL participated in study design, oversaw data collection during both phases of research and provided feedback on the manuscript. AK helped manage data collection and reviewed the manuscript. RW designed the initial study, supervised and participated in phase 1 data collection, and provided feedback on the manuscript. All authors reviewed and approved the final manuscript.
